# CEH-20/Pbx and UNC-62/Meis function upstream of *rnt-1*/Runx to regulate asymmetric divisions of the *C. elegans* stem-like seam cells

**DOI:** 10.1242/bio.20134549

**Published:** 2013-06-06

**Authors:** Samantha Hughes, Charles Brabin, Peter J. Appleford, Alison Woollard

**Affiliations:** Department of Biochemistry, University of Oxford, South Parks Road, Oxford OX1 3QU, UK

**Keywords:** *C. elegans*, Seam cell, Asymmetric cell division, *ceh-20*/Pbx, *unc-62*/Meis, *rnt-1*/Runx

## Abstract

*Caenorhabditis elegans* seam cells divide in the stem-like mode throughout larval development, with the ability to both self-renew and produce daughters that differentiate. Seam cells typically divide asymmetrically, giving rise to an anterior daughter that fuses with the hypodermis and a posterior daughter that proliferates further. Previously we have identified *rnt-1* (a homologue of the mammalian cancer-associated stem cell regulator Runx) as being an important regulator of seam development, acting to promote proliferation; *rnt-1* mutants have fewer seam cells whereas overexpressing *rnt-1* causes seam cell hyperplasia. We isolated the interacting CEH-20/Pbx and UNC-62/Meis TALE-class transcription factors during a genome-wide RNAi screen for novel regulators of seam cell number. Animals lacking wild type CEH-20 or UNC-62 display seam cell hyperplasia, largely restricted to the anterior of the worm, whereas double mutants have many additional seam cells along the length of the animal. The cellular basis of the hyperplasia involves the symmetrisation of normally asymmetric seam cell divisions towards the proliferative stem-like fate. The hyperplasia is completely suppressed in *rnt-1* mutants, and *rnt-1* is upregulated in *ceh-20* and *unc-62* mutants, suggesting that CEH-20 and UNC-62 function upstream of *rnt-1* to limit proliferative potential to the appropriate daughter cell. In further support of this we find that CEH-20 is asymmetrically localised in seam daughters following an asymmetric division, being predominantly restricted to anterior nuclei whose fate is to differentiate. Thus, *ceh-20* and *unc-62* encode crucial regulators of seam cell division asymmetry, acting *via rnt-1* to regulate the balance between proliferation and differentiation.

## Introduction

Asymmetric cell divisions provide an important mechanism for the generation of cellular diversity during development and tissue regeneration. Furthermore, misregulation of asymmetric divisions has been associated with carcinogenesis, underscoring the biomedical importance of understanding this process (Knoblich, 2010; Neumüller and Knoblich, 2009). There are many examples of asymmetric divisions in biology, but one of the most notable is that of a stem cell, which characteristically produces one daughter that adopts a differentiated fate and another that remains a stem cell and proceeds to proliferate further (Knoblich, 2008). Since the discovery that multiple cell types may be derived from single self-renewing stem cells, the potential of these cells to generate all tissue types has led to intensive research into their unique properties.

In *C. elegans* the neuroectodermal seam cells provide a useful model for stem cell regulation. During larval development, they undergo reiterative asymmetric divisions in order to both self-renew and differentiate into epidermal cells, neurons, glia and ray precursor cells of the male tail. Worms hatch with 10 seam cells on each side (H0, H1, H2, V1–6 and T). The general pattern of division involves an asymmetric division at each larval stage, producing a posterior daughter that retains the ability to divide further and an anterior daughter that adopts a differentiated fate, most commonly contributing to epidermal tissue by fusing with the hyp7 syncytium (Sulston and Horvitz, 1977). In addition, there is a single symmetrical division at L2 in the V lineage, where both daughter cells retain proliferative ability and consequently expand the pool of seam cells so that adult hermaphrodites have 16 seam nuclei per side.

We, and others, have previously shown that the Runx transcription factor *rnt-1* and its DNA binding partner *bro-1* (a homologue of the Runx binding factor CBFβ) are crucial to regulate the balance between seam cell proliferation and differentiation, acting to promote the proliferative fate in posterior seam daughters (Kagoshima et al., 2007; Nimmo et al., 2005; Xia et al., 2007). Thus, mutations in *rnt-1* or *bro-1* reduce the number of seam cells due to failures in particular seam cell divisions, whereas overexpressing these genes leads to seam cell hyperplasia at the expense of other differentiated cell types (Kagoshima et al., 2007; Nimmo et al., 2005). Strikingly, Runx and CBFβ proteins in other organisms are also important for balancing proliferation and differentiation, particularly in stem cell lineages, for example during haematopoiesis (Blyth et al., 2009; Okuda et al., 1996; Okumura et al., 2007). This underscores the applicability of the seam cell system for understanding stem cell biology and the usefulness of using seam cell number as an assay for identifying genes that are required to control the balance between cell proliferation and differentiation. In particular, *rnt-1* and *bro-1* are the solo homologues of Runx and CBFβ, therefore studying the molecular pathways involving these genes in *C. elegans* is not confounded by the redundancy issues experienced in other model systems.

RNT-1 and BRO-1 are required for divisions throughout larval development, and are likely to interact directly with cell cycle regulators (Kagoshima et al., 2007; Nimmo et al., 2005; Xia et al., 2007). One recently identified direct regulator of *bro-1* is the GATA factor ELT-1, which has dual roles in both promoting proliferation (*via bro-1*) and inhibiting differentiation (*via* the fusogen *eff-1*) (Brabin et al., 2011). Other regulators (and targets) of *rnt-1* and *bro-1* are yet to be identified.

Wnt signalling has been shown to be essential for establishing seam cell division asymmetry, in common with its role in a variety of organisms (Clevers, 2006; Grigoryan et al., 2008; Hayden et al., 2007; Yamamoto et al., 2011). In *C. elegans*, a variant of Wnt signalling termed the Wnt/β-catenin asymmetry pathway regulates asymmetric divisions throughout seam cell development (reviewed by Mizumoto and Sawa, 2007b) and is thought to act in parallel to the *rnt-1/bro-1* pathway (Gleason and Eisenmann, 2010). Asymmetry in the dividing mother cell is established by the β-catenin WRM-1, which is enriched at the anterior cortex during telophase *via* a microtubule-dependent mechanism (Sugioka et al., 2011), thereby excluding WRM-1 from the anterior daughter nucleus (Mizumoto and Sawa, 2007a; Nakamura et al., 2005; Takeshita and Sawa, 2005). WRM-1 in the posterior daughter nucleus causes the export of the TCF/LEF homologue POP-1, thus setting up nuclear reciprocal asymmetry between these two factors (Lo et al., 2004; Nakamura et al., 2005; Rocheleau et al., 1999). The further reciprocal asymmetry between POP-1 and the β-catenin SYS-1 leads to the subsequent transcriptional activation of target genes in the posterior (signalled) daughter and repression of target genes in the anterior daughter, thus establishing the developmental fate appropriate to each daughter (Calvo et al., 2001; Huang et al., 2007; Lin et al., 1998; Lo et al., 2004; Phillips et al., 2007; Rocheleau et al., 1997; Shetty et al., 2005). The engrailed homologue *ceh-16* is also required to control seam cell number, and is thought to act in the Wnt pathway. Perturbation of Wnt components suppresses the seam defects associated with *ceh-16* mutants, suggesting that *ceh-16* may act as an upstream regulator of the Wnt pathway (Huang et al., 2009).

Here, we describe a genome wide RNAi screen to identify novel regulators of seam cell divisions. Two genes, *ceh-20* and *unc-62*, encoding interacting Pbx and Meis transcription factors, were isolated as a result of their knockdown phenotype causing significant seam cell hyperplasia, resulting from the symmetrisation of divisions that are normally asymmetric. Epistasis analysis suggests that *ceh-20* and *unc-62* act upstream of *rnt-1* to repress inappropriate seam cell proliferation in daughters destined to differentiate. Thus, our studies show that CEH-20 and UNC-62 are two novel components of the molecular circuitry controlling seam cell divisions. It is striking that all the factors we have identified thus far as being crucial for the correct coordination of proliferation and differentiation in *C. elegans* seam cells have human homologues that are implicated in carcinogenesis, particularly acute myeloid leukaemia (Blyth et al., 2005; Cameron and Neil, 2004; Geerts et al., 2005; Licht, 2001; Mikesch et al., 2007; Osato, 2004; Wildonger and Mann, 2005; Wong et al., 2007).

## Materials and Methods

### Strains and maintenance of worms

Strains were derived from the wild type N2 Bristol strain and maintained at 20°C as described previously (Brenner, 1974). Strains used are detailed in supplementary material Table S1.

### Microscopy

DIC (Nomarski) and fluorescent imaging was carried out using a Zeiss AxioSKOP2 microscope with a Zeiss AxioCamMR digital camera. Photomicrographs were taken using a ×63 oil immersion objective (Zeiss) and Axiovision software (Release 4.5). Images of whole worms were compiled using Adobe Photoshop 7.0 and the backgrounds merged. Confocal images were taken on a Leica TCS SP5II using Leica Application Suite Advanced Fluorescence Lite software (Release 2.2.1). In both cases, animals were mounted on agarose pads (2% agarose, 0.5% 1-phenoxy-2-propanol in M9) in 0.2% 1-phenoxy-2-propanol.

### Lineage analysis

Lineage analysis was performed as described previously (Sulston and Horvitz, 1977). Seam nuclei were distinguished from hypodermal nuclei based on their morphology in addition to the expression of the seam specific GFP reporter, *scm::gfp*. Microscopy was performed with Normarski (DIC) optics and a ×100 oil immersion objective (Zeiss) using a Zeiss AxioPlan microscope.

### RNAi

RNAi was performed using the Ahringer RNAi library and protocol (Kamath and Ahringer, 2003). dsRNA was delivered by feeding to L4 stage *JR667* animals which carry the integrated seam specific *scm::gfp* marker (Koh and Rothman, 2001). For experiments involving the *wrm-1(ne1982*ts) strain *EW95*, L4 animals were placed onto RNAi plates at 15°C. The parents were removed once eggs were laid (approximately 32 hours) and the plates transferred to the restrictive temperature (26.5°C). In all experiments, seam cell number was counted in L4 offspring. Control RNAi was performed using *HT115* bacteria transformed with an empty *L4440* vector.

The *unc-62* feeding clone was not available in the library and was constructed separately by PCR amplification from cDNA using primer pair 1 (supplementary material Table S2). A 1.4 kb fragment was sub-cloned into the Fire Lab RNAi vector *L4440* (Timmons and Fire, 1998). The *unc-62* RNAi clone was verified by sequencing prior to transformation into *HT115* bacteria.

### *ceh-20* cDNA::GFP translational reporter construction

*ceh-20::gfp* translational reporter constructs were obtained by fusion PCR (Hobert, 2002). *ceh-20* with a GFP tag was amplified from cDNA using primer pair 2 (supplementary material Table S2). In parallel, the GFP ORF was amplified from the Fire Lab vector *pPD95.75* with primer pair 3. These PCR fragments were used as a template for fusion PCR with primer pair 4. The resulting PCR product was cloned into *pCR2.1*®-XL-TOPO® (Invitrogen). *pAW524* consisted of *ceh-20::gfp* from wild type animals while the *ceh-20(ay9)::gfp (pAW547)* and *ceh-20(mu290)::gfp* (*pAW531*) constructs were generated using cDNA from the respective mutant animals.

### *ceh-20* promoter driven cDNA::GFP translational reporters

The 2180 bp endogenous *ceh-20* promoter was amplified from genomic DNA (primer pair 5, supplementary material Table S2). The fragment was sub-cloned into the Fire Lab vector *pPD49.26* (*pAW530*). The *ceh-20::gfp* plasmids (*pAW524*, *pAW531* and *pAW547*) were digested with *Spe*I and *EcoR*V ligated into *pAW530*, previously digested with *Nhe*I and *EcoR*V. Thus *ceh-20p::ceh-20*(WT)::*gfp* (*pAW532*), *ceh-20p::ceh-20(mu290)::gfp* (*pAW536*) and *ceh-20p::ceh-20(ay9)::gfp* (*pAW550*) were generated.

### Seam specific GFP translational reporters

To drive specific expression in the seam, the *bro-1CNE* (*pAW362*) was used, which comprises the *bro-1* conserved non-coding element (CNE) and *pes-10* minimal promoter (Brabin et al., 2011). Plasmids *pAW524*, *pAW531* and *pAW547* were digested using *Spe*I and *EcoR*V and cloned into *pAW362*, previously digested with *Nhe*I and *EcoR*V, generating *pAW538* (*bro-1CNE*::*ceh-20(WT)::gfp*), *pAW537* (*bro-1CNE*::*ceh-20(mu290)::gfp*) and *pAW551* (*bro-1CNE*::*ceh-20(ay9)::gfp*).

### Site directed mutagenesis

Amino acid 191 within NLS I of *ceh-20* was converted from a lysine (AAA) to a proline (CCG). In this instance, DNA was PCR amplified from *pAW538* using primer pair 6 generating *pAW588 (bro-1CNE::ceh-20(*mutated NLS I*)::gfp*). The same primers were used to amplify DNA from *pAW537* to generate *pAW589* (*bro-1CNE*::*ceh-20*(mutated NLS I+*mu290)::gfp*).

### Construction of transgenic worms

Plasmids were injected into the syncytial gonad of L4 hermaphrodite animals at a concentration of 10–20 ng/µl as described previously (Mello and Fire, 1995).

### Electrophoretic Mobility Shift Assay plasmids

PCR was used to amplify the full length *ceh-20* and *unc-62* cDNA using primer pairs 7 and 8 respectively (supplementary material Table S2) and cloned into pCR®-XL-TOPO® (Invitrogen) resulting in plasmids *pAW521* (*ceh-20*) and *pAW579* (*unc-62*). The *TNT*® Quick Coupled Transcription/Translation kit (Promega) was used for *in vitro* transcription and translation of cDNA constructs. The probe of a consensus Pbx/Meis binding site 5′-CGGAGGACCCGTGATTGACAGGTTCGCAGTGAT-3′ and 5′-CATCACTGCGAACCTGTCAATCACGGGTCCTCC-3′ (Shanmugam et al., 1999) was labelled with [γ-^32^P]ATP using polynucleotide kinase (Promega). The oligos were annealed by heating at 95°C for 5 minutes followed by gradual cooling to room temperature. The DNA binding reaction was carried out on ice for 30 minutes in Ficoll 400 (20% w/v) with PolydI/dC (1 mg/ml). Reactions were run on a 7% non-denaturing polyacrylamide gel at 4°C in 0.5×TBE.

### Real-time quantitative PCR analysis

RNA was extracted from L4 synchronised larvae using the hot phenol method (Furger et al., 2001). mRNA levels of *rnt-1* and *bro-1* (primer pairs 9 and 10, supplementary material Table S2) and a housekeeping gene *nuo-2* (primer pair 11) were measured using GoTaq® qPCR Master Mix (Promega) with a StepOnePlus^TM^ Real-Time PCR System (Applied Biosystems). The Ct values of *rnt-1* and *bro-1* were measured in all strains and normalised to *nuo-2*, an NADH ubiquinone oxidoreducatase expressed in all seam cells, to correct for seam cell number. Expression levels were assayed using the ΔΔCt method (Schmittgen and Livak, 2008).

## Results

### A genome-wide RNAi screen identified *ceh-20* as a regulator of seam cell proliferation

**Fig. 1.**
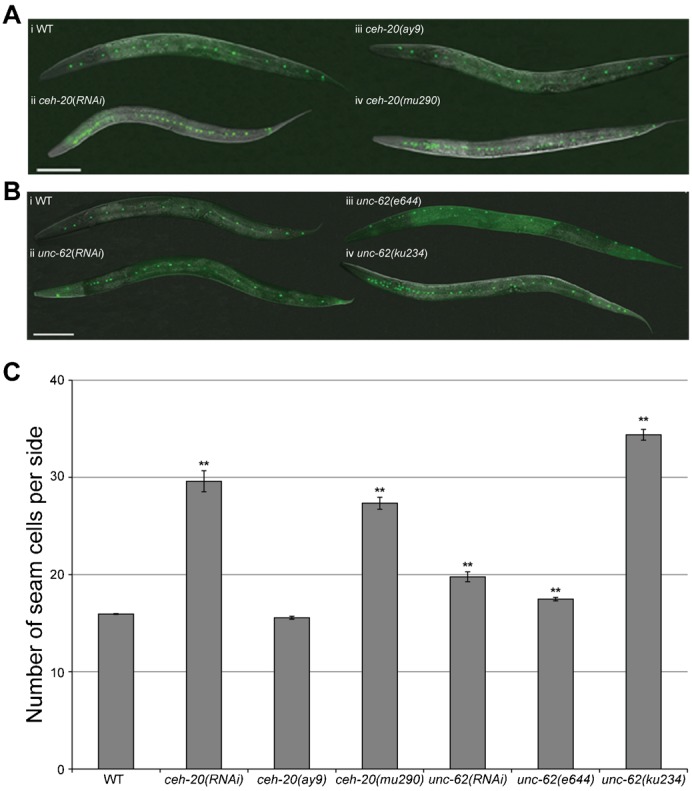
Seam cell hyperplasia results from loss of *ceh-20* and *unc-62* function. (**A**) (i) Wild type late L4 animal carrying the integrated seam cell marker, *scm::gfp* (strain *JR667*), with 16 seam cells per side. (ii) *scm::gfp; ceh-20(RNAi*) animals display seam cell hyperplasia, with an anterior bias, as identified in our genome-wide screen. (iii) *ceh-20(ay9)*; *him-5(e1490) scm::gfp* (strain *AW413*) animals do not display seam cell hyperplasia. (iv) *ceh-20(mu290*); *scm::gfp* (strain *AW417*) worms have seam cell hyperplasia, again displaying a strong anterior bias. (**B**) (i) Wild type animal carrying the *scm::gfp* marker (strain *JR667*) with 16 seam cells. (ii) *scm::gfp; unc-62(RNAi)* animal exhibiting modest seam cell hyperplasia. (iii) *unc-62(e644)*; *him-5(e1490*) *scm::gfp* (strain *AW392*) animals displaying low levels of seam cell number. (iv) *unc-62(ku234)*; *him-5(e1490) scm::gfp* (strain *AW673*) mutants display severe seam cell hyperplasia which is predominantly localised to the head region of the animal. In all images anterior is to the left and dorsal is at the top. Scale bars: 100 µm. (**C**) Graph indicating the seam cell numbers in the strains described above (*n*>60 in all cases). Error bars represent s.e.m. and ** indicates the 2-sample t-test where each strain was compared to the wild type, where *P*<0.01.

**Fig. 2.**
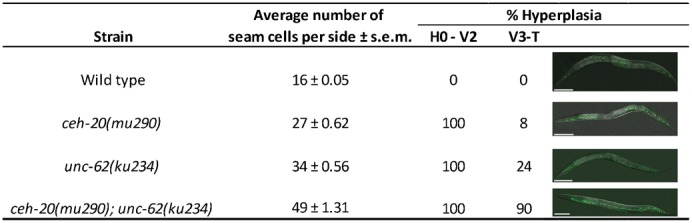
*ceh-20* and *unc-62* single mutants display seam cell hyperplasia with an anterior bias. The hyperplasia observed in *ceh-20* and *unc-62* single mutant animals (strains *AW417* and *AW673*) is largely restricted to the anterior region of the animal. In contrast, 90% of the double mutant animals (strain *AW679*) displayed significantly more seam cells in the posterior region as well as the anterior, resulting in full body hyperplasia (*n* = 100 for each strain analysed). Scale bars: 20 µm.

A genome-wide RNAi by feeding screen was undertaken to identify novel regulators of seam cell proliferation, using animals carrying the integrated *scm::gfp* seam-specific marker (strain *JR667*) (Koh and Rothman, 2001). Seam cell number was counted in late L4, after the final asymmetric division but before terminal differentiation. We identified 307 genes that when silenced by RNAi, altered seam cell number in L4 animals (Table 1; supplementary material Table S3). Of these, 137 genes increased the number of seam cells while the remainder reduced the number of seam cells to below 16. The most striking phenotype observed was the seam cell hyperplasia caused by *ceh-20* knockdown. Seam cell number is approximately doubled in *ceh-20(RNAi)* animals (Fig. 1A,C). Two viable *ceh-20* mis-sense alleles (Jiang et al., 2009; Takács-Vellai et al., 2007; Yang et al., 2005) were next tested for seam cell hyperplasia and *mu290*, but not the weaker allele *ay9*, displayed an increase in seam cell number, comparable to that observed with RNAi (Fig. 1A,C). In both cases (*mu290* and *ceh-20(RNAi)*), seam hyperplasia showed a strong anterior bias in all animals observed (Fig. 1A, Fig. 2).

**Table 1.**
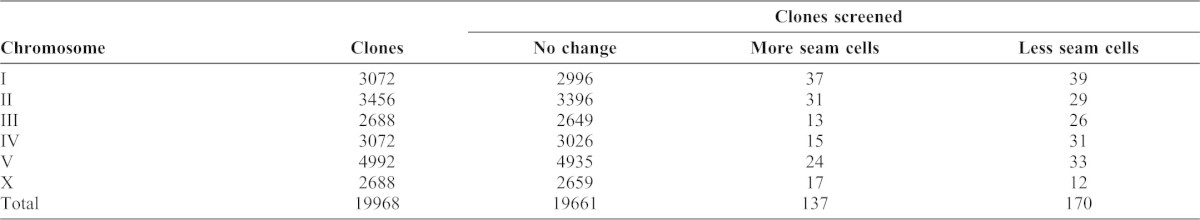
Results of the whole genome RNAi screen. The table displays the number of clones screened per chromosome, and overall changes in seam cell number observed by counting *scm::gfp* positive nuclei in late L4 animals. Overall, 1.5% of genes were found to affect seam cell number when silenced by RNAi (see also supplementary material Table S3).

### The CEH-20 binding partner UNC-62 is also essential for limiting seam cell proliferation

CEH-20 is a member of the conserved Pbx family of transcriptional regulators, initially identified as human leukaemic proto-oncogenes (Laurent et al., 2008), that are known to bind cooperatively to Meis proteins *via* interaction of their conserved PBC domains with the Meis Homothorax-Meis (HM) domain (Abu-Shaar et al., 1999; Rieckhof et al., 1997; Ryoo et al., 1999; Stevens and Mann, 2007). Pbx proteins are members of the Three Amino acid Loop Extension (TALE) class of homeodomain proteins, which have been shown to be important transcription factors or transcriptional co-factors in development throughout the animal kingdom (Moens and Selleri, 2006). *C. elegans* has two members of the Meis class of TALE proteins, *psa-3* and *unc-62* (Arata et al., 2006; Van Auken et al., 2002). We found no change in overall seam cell number following *psa-3* RNAi (data not shown).

*unc-62* was not isolated in our screen because it is not present in the RNAi feeding library. We therefore constructed an *unc-62* RNAi feeding clone that caused a modest elevation in seam cell number when fed to worms (Fig. 1B,C). The mutant allele *ku234* gave a much stronger phenotype while a second mutant allele, *e644*, did not have any effect on seam cell number (Fig. 1B,C). The hyperplasia in *ku234* animals was again characterized by an obvious anterior bias, similar to that of *ceh-20(mu290)* and *ceh-20(RNAi)* animals (Fig. 2). *unc-62* is subject to alternative splicing (Van Auken et al., 2002). The *ku234* allele results in a point mutation in the start codon of the 1b transcript. This has been suggested to shift the start codon downstream, likely resulting in a truncated protein lacking the important Pbx-interacting HM domain (Van Auken et al., 2002). The *e644* allele introduces a stop codon in exon 7b, which may disrupt the homeodomain (Van Auken et al., 2002). It is thus not clear at present why seam hyperplasia is observed in *ku234* but not *e644* animals. Given the relative ineffectiveness of *unc-62* RNAi, subsequent experiments utilized the *ku234* allele. In the case of *ceh-20*, RNAi was very robust and often more convenient to use than the *mu290* allele.

The *ceh-20; unc-62* double mutant, strain *AW679*, displayed much more extensive seam hyperplasia, up to 70 seam cells per side, extending throughout the length of the animal (Fig. 2), suggesting that CEH-20 and UNC-62 interact in a complex to control the correct development of the seam lineage. This mirrors the function of CEH-20/UNC-62 in other tissues, for example the M lineage and the vulva (Jiang et al., 2009; Yang et al., 2005).

### Regulatory interactions between CEH-20 and UNC-62

**Fig. 3.**
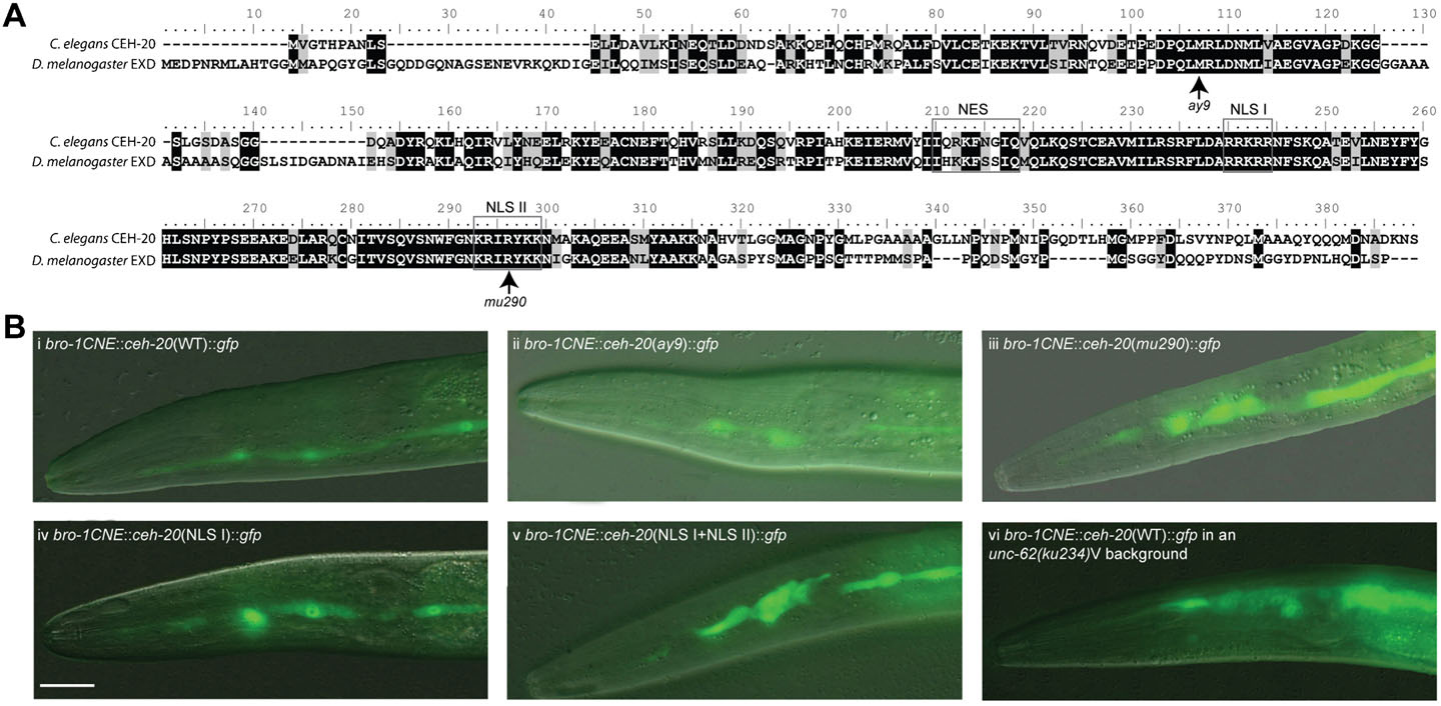
CEH-20 displays dynamic sub-cellular localisation, which is UNC-62 dependent. (**A**) Alignment of CEH-20 from *C. elegans* and the *D. melanogaster* homologue Extradenticle, Exd, showing the lesions in *ay9* (M78I) and *mu290* (R245H) alleles. The grey boxes indicate the nuclear export sequence (NES) and the two nuclear localisation sequences (NLS). (**B**) Expression pattern of *ceh-20 cDNA::gfp* constructs under control of a seam specific promoter (*bro-1CNE*). (i) WT *ceh-20::gfp* (strain *AW555*), showing predominantly nuclear expression in seam cells. (ii) *ay9 ceh-20::gfp* (strain *AW550*) displaying a similar expression pattern. (iii) *mu290 ceh-20::gfp* (strain *AW541*), showing much greater cytoplasmic expression of *ceh-20.* (iv) *ceh-20::gfp* containing a mutation in NLS I (strain *AW593*). In this case, the expression pattern is similar to WT. (v) *ceh-20::gfp* containing mutations in both NLS I and NLS II (strain *AW601*), with predominantly cytoplasmic expression. (vi) WT *ceh-20::gfp* in an *unc-62(ku234)* background (*AW564*), again showing high levels of cytoplasmic *ceh-20*. In all images anterior is to the left and dorsal is at the top. Scale bar: 20 µm.

UNC-62 has been shown to bind to CEH-20 in a yeast 2 hybrid assay (Jiang et al., 2009), consistent with previously described Meis/Pbx interactions in other systems (Abu-Shaar et al., 1999; Arata et al., 2006; Potts et al., 2009; Rieckhof et al., 1997; Ryoo et al., 1999; Van Auken et al., 2002). To confirm this interaction we performed an Electrophoretic Mobility Shift Assay (EMSA), which demonstrated binding to the consensus Pbx/Meis DNA binding site by a complex of CEH-20 and UNC-62 (supplementary material Fig. S1). In addition, Meis proteins have previously been shown to be required for the nuclear localisation of Pbx (Abu-Shaar et al., 1999; Arata et al., 2006; Potts et al., 2009; Rieckhof et al., 1997; Ryoo et al., 1999; Stevens and Mann, 2007). We therefore examined the sub-cellular localisation of CEH-20 in seam cells using *ceh-20::gfp* constructs driven by the strong seam enhancer *bro-1*CNE (Brabin et al., 2011). Overall, we observed that wild type *ceh-20* constructs were expressed predominantly in seam nuclei (Fig. 3B). However, a construct containing the *mu290* mutation (associated with seam hyperplasia) was strongly enriched in the cytoplasm (Fig. 3B). The *mu290* mutation affects one of the two nuclear localisation signals (NLSs) in *ceh-20*, and our analysis demonstrated that this NLS (NLS II, but not NLS I) is essential for correct nuclear localisation in seam cells (Fig. 3B). Enriched cytoplasmic localisation was also observed in *unc-62(ku234)* mutants, indicating that UNC-62 is indeed required for nuclear enrichment of CEH-20 in seam cells (Fig. 3B).

### The seam cell hyperplasia in *ceh-20* and *unc-62* mutants is a consequence of symmetrisation of seam cell divisions

**Fig. 4.**
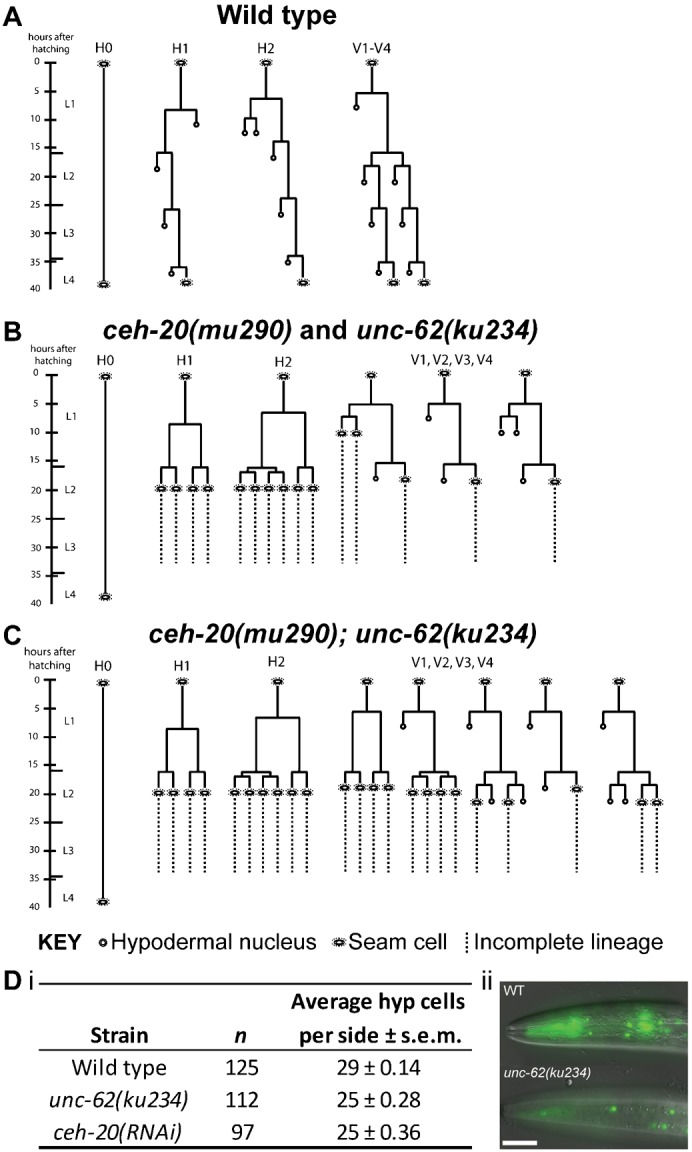
Lineage analysis of *ceh-20* and *unc-62* single and double mutants. (**A**) Lineage trace of wild type hermaphrodite, from hatching to L4, in H0, H1, H2 and V1–4 seam lineages. Note that the L1 division of H1 has reversed polarity compared with V lineage L1 divisions, and that the L1 division of H2 is symmetrical. (**B**) Lineage trace showing representative division defects (from 10–15 lineaged animals per strain) in *ceh-20(mu290)* and *unc-62(ku234)* single mutants from hatching to L3; the most obvious aspect of the phenotype is observed in the head lineages (H1 and H2), where the symmetrisation of divisions to the seam fate results in the over-proliferation of seam cells. Defects in V1–4 divisions are more variable, but often include the transformation of the L2 division from a symmetric division to an asymmetric division and the inappropriate division of Vn.a. (**C**) Representative lineage trace of *ceh-20(mu290)*; *unc-62(ku234)* double mutant animals (*n* = 10), in which symmetrisation events are observed in the V as well as the H lineages. (**D**) (i) With an increase in seam cell number, the number of seam derived hypodermal nuclei significantly decreases. Wild type animals carrying *dpy-7*p::*yfp* (strain *AW525*) have 29–30 hypodermal nuclei derived from the seam lineages (H, V and T) on the ventral side. In *unc-62(ku234)* (strain *AW682*) and *dpy-7*p*::yfp*; *ceh-20(RNAi)* animals, the number of nuclei is significantly reduced (*P*<0.01). (ii) Wild type (top panel) and *unc-62(ku234)* animals (bottom panel) containing the *dpy-7::yfp* reporter, showing that the latter exhibit reduced numbers of hypodermal nuclei in the head. Scale bar: 50 µm.

To investigate the cellular basis of the *ceh-20* and *unc-62* hyperplasia phenotypes, lineage analysis was performed on wild type and mutant strains (Fig. 4). In both single mutants, additional seam cells first appeared at the L1 division (Fig. 4B), with the normally asymmetric division of H1 being symmetrised towards the proliferative fate (the H2 L1 division is symmetric in wild type animals). The seam identity of these daughters was established by the fact that they continued to express *scm::gfp* whereas non seam daughters usually lose their GFP within one or two hours of the L1 division (data not shown). Both daughters of H1 and H2 were seen to divide again, in the symmetric pattern, at the L2 stage. Significantly, the H2.a daughter did not divide several hours before the L2 division, as is normal, but instead delayed the timing of its division to coincide with that of other seam daughters at L2, suggesting a complete conversion of developmental fate to that of the posterior daughter. These defects occurred in all animals observed. Lineages were not followed beyond L2 because there were simply too many seam cells to observe.

While the hyperplasia in the single *ceh-20* and *unc-62* mutants is most striking in the H1 and H2 lineages, the V lineages were also affected, but with much more variable outcomes (Fig. 4B). Here, early Vn divisions (during L1) were sometimes symmetrised towards the proliferative fate, but Vn.a divisions were also observed to be symmetrised towards the hypodermal (differentiative) fate. We also often observed the Vn.p daughter undergoing an asymmetric division at the beginning of L2, instead of the normal symmetric division. Thus, in the posterior of the worm, one lineage might produce more cells with the seam fate than normal, resulting in clusters of extra seam cells, whereas others could produce fewer, producing gaps in the seam.

In the double mutant, however, extensive symmetrisation events were observed throughout H and V lineages (Fig. 4C), especially after L1, leading to much more severe hyperplasia along the length of the worm (Fig. 2).

The most common outcome of an asymmetric division in the seam lineage is a single seam daughter plus a differentiated hypodermal daughter that expresses *dpy-7* and fuses with hyp7. Therefore, the abnormal expansion of seam cell number would be expected to occur at the cost of the hypodermal fate. To confirm this, we used an integrated *dpy-7p::yfp* reporter and found significantly fewer DPY-7::YFP positive nuclei in *ceh-20* and *unc-62* mutants compared to wild type (Fig. 4D).

### *ceh-20* and *unc-62* function downstream of or in parallel to the Wnt pathway to regulate seam divisions

**Fig. 5.**
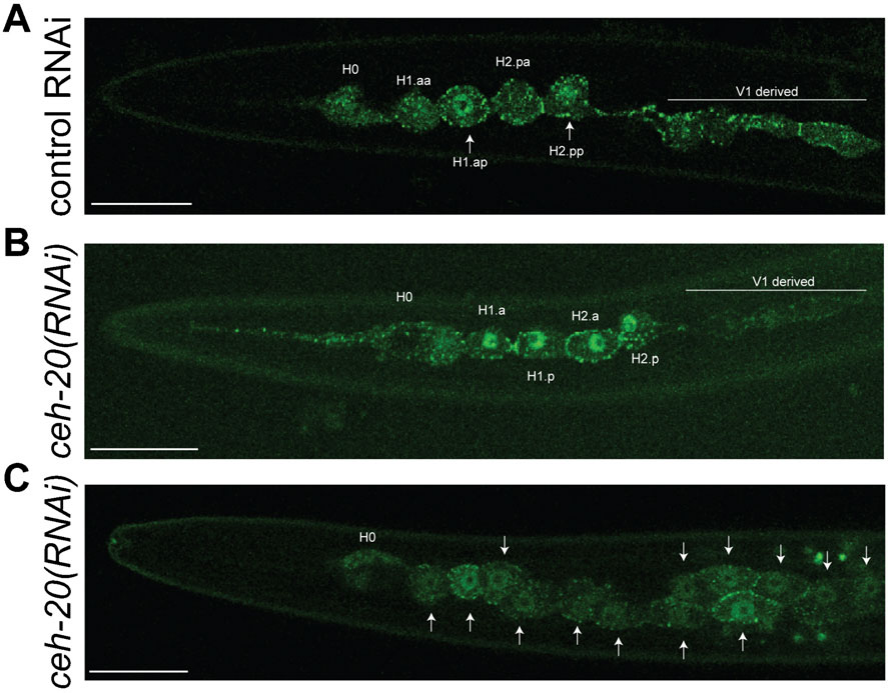
WRM-1 distribution is perturbed in the absence of CEH-20. The anterior seam cells of animals expressing *wrm-1::gfp* (strain *HS1417*). (**A**) Representative control animal exposed to *L4440* RNAi feeding bacteria observed at the L2 asymmetric (late) division. Asymmetric nuclear distribution of WRM-1::GFP is observed, with WRM-1 enriched in posterior daughter nuclei destined to proliferate further (arrows). V1 derived nuclei are out of the focal plane in this image. (**B**) During the L2 symmetrical (early) division in *wrm-1::gfp; ceh-20(RNAi)* animals, WRM-1::GFP is localised to both anterior and posterior daughter nuclei. (**C**) *wrm-1::gfp; ceh-20(RNAi)* animals display seam cell hyperplasia with WRM-1::GFP localised to the nuclei of all cells (arrows). In all images anterior is to the left and dorsal is at the top. Scale bars: 10 µm. Data shown is representative of all animals imaged (*n*>30).

Given the well-characterised role of Wnt signalling in regulating asymmetric seam cell divisions, we tested possible interactions between *ceh-*20, *unc-62* and the Wnt pathway. In wild type animals, the β-catenin WRM-1 has been shown to be asymmetrically localised during asymmetric V5 and T seam cell divisions, being enriched in posterior daughter nuclei and at the anterior cortex (Mizumoto and Sawa, 2007a; Takeshita and Sawa, 2005) This is thought to be essential for correct cell fate patterning, as WRM-1 in the nucleus causes the export of POP-1/TCF and thus subsequent adoption of the signalled fate (which is proliferative in the case of the seam cells) (Calvo et al., 2001; Gleason and Eisenmann, 2010; Herman, 2001; Huang et al., 2007; Lin et al., 1998; Lo et al., 2004; Phillips et al., 2007; Shetty et al., 2005). Using a *wrm-1::gfp* reporter (strain *HS1417*), we confirmed that WRM-1 asymmetric localisation also occurs in anterior seam cell lineages (H1 and H2) during the L3 asymmetric division (Fig. 5A).

In contrast, *ceh-20(RNAi)* animals displayed distinct nuclear WRM-1::GFP localisation in all daughters (Fig. 5B,C). An important question, however, is whether abnormal WRM-1 localisation in *ceh-20(RNAi)* animals causes the symmetrisation of seam divisions (implying that *ceh-20* works upstream of WRM-1), or whether it is simply an indirect effect of earlier perturbations in cell fate. In order to distinguish between these possibilities, we performed *ceh-20* RNAi in a *wrm-1 ts* mutant background (using the *wrm-1*(*ne1982*ts) allele). No suppression of hyperplasia was observed at the restrictive temperature (Table 2), thus we conclude that *ceh-20* (and, by association, *unc-62*) function in a parallel pathway (or downstream of WRM-1) to control seam cell proliferation.

**Table 2.**
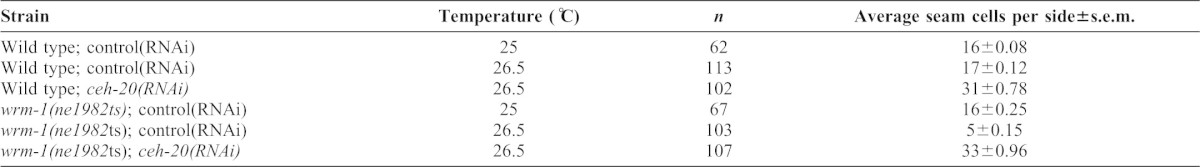
Seam hyperplasia is not dependent upon the presence of WRM-1. At both the permissive (25°C) and restrictive temperature (26.5°C) wild type animals (strain *JR667*) have 16 seam cells per side, whereas *wrm-1 (ne1982ts); scm::gfp* (strain *EW95*) animals have just 5 seam cells on average at the restrictive temperature. However, seam cell hyperplasia is evident to the same extent in WT and *wrm-1 ts* mutants following exposure to *ceh-20* RNAi at the restrictive temperature.

**Fig. 6.**
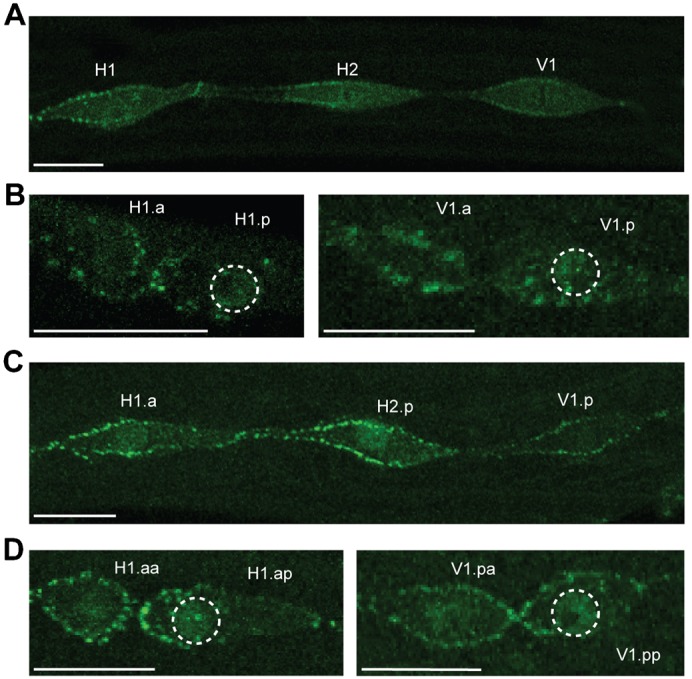
WRM-1 is asymmetrically distributed at all seam divisions, regardless of division mode. (**A**) At telophase of the L1 division in WT animals, WRM-1::GFP can be clearly seen at the anterior cortex of dividing seam cells, even in those that are set to divide symmetrically (H2) or with reversed polarity (H1). (**B**) Just after division, WRM-1 is enriched at the posterior nucleus and anterior cortex in both the H1 and V1 daughter cells, even though H1.p differentiates. (**C**) WRM-1::GFP is again concentrated at the anterior cortex of the seam cells at telophase of the L2 division, in H1.a and H2.p (about to divide asymmetrically at this stage) as well as in V1.p (about to divide symmetrically). (**D**) Immediately following this division, WRM-1 is localized to the anterior cortex and posterior nuclei of H1.a daughters and V1.p daughters, even though both daughters of V1.p retain the proliferative fate. Scale bars: 10 µm.

Interestingly, we also observed that WRM-1 distribution does not always follow the predicted pattern during certain seam cell divisions. For example, in wild type, the asymmetric division of H1 at L1 is reversed compared with Vn cells, with the anterior daughter retaining the seam fate and the posterior daughter fusing with hyp7 (Fig. 4). Thus, we would expect WRM-1 to be enriched in the anterior nucleus and posterior cortex. However, we found WRM-1 to be predominantly localised to the anterior cortex and posterior daughter nucleus, just as it is in V lineages (Fig. 6A,B). Similarly, WRM-1 was also asymmetrically distributed in H2 at L1 and in the Vn.p cells at the first L2 division, even though these divisions are normally symmetrical (Fig. 6C,D). Thus, WRM-1 distribution does not always appear to correlate with subsequent cell fate determination during seam cell divisions.

### *ceh-20* and *unc-62* act upstream of *rnt-1/bro-1* to prevent inappropriate seam cell proliferation

**Fig. 7.**
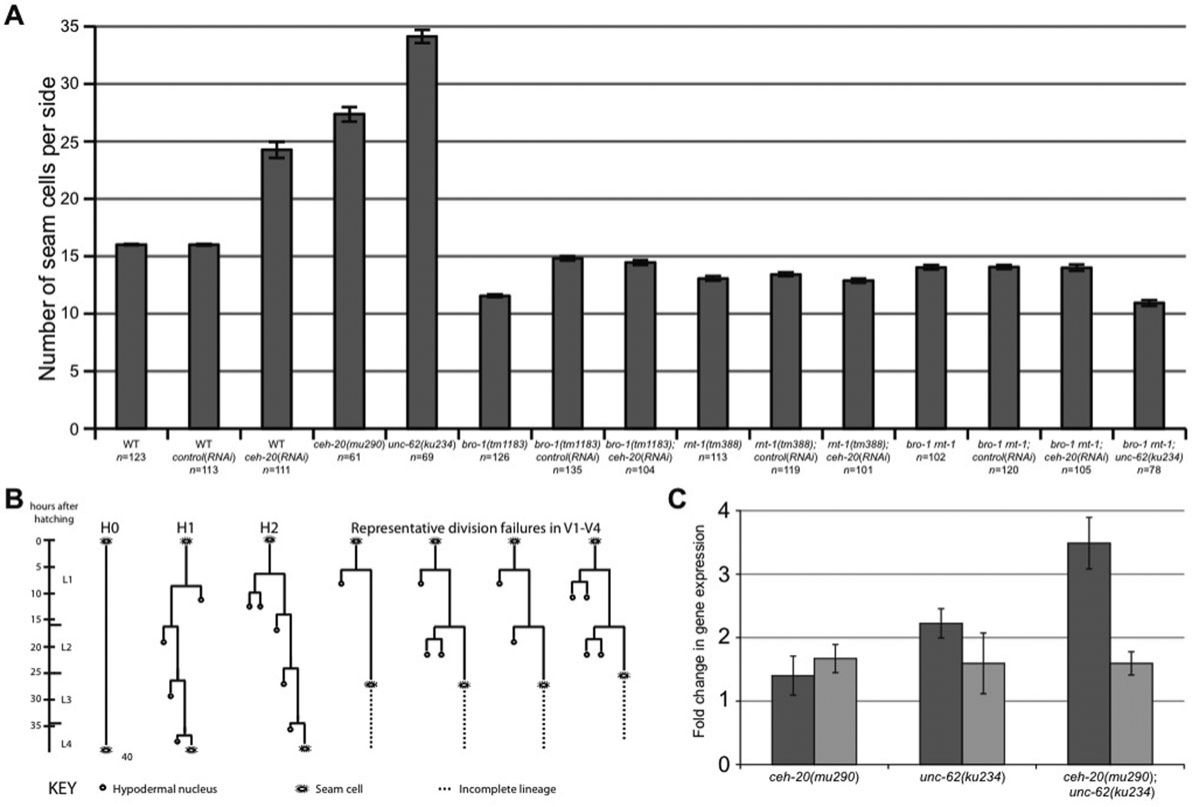
*ceh-20* and *unc-62* associated seam hyperplasia is dependent on *rnt-1*. (**A**) Seam cell counts as assayed using the *scm::gfp* reporter strain *JR667*. Strain details and *n* numbers are listed on the x-axis. Overall, *ceh-20/unc-62* associated seam cell hyperplasia is completely suppressed in *rnt-1* and *bro-1* mutants. Error bars represent s.e.m. (**B**) Representative lineage trace of *bro-1(tm1183) rnt-1(tm388); unc-62(ku234)* animals (strain *AW674*) showing complete suppression of the H1 and H2 symmetrisation events normally associated with *unc-62* single mutants. Data shown is representative of 8 animals lineaged. (**C**) Real-time quantitative PCR analysis of *rnt-1* transcript levels (dark grey bars) and *bro-1* transcript levels (light grey bars) in *ceh-20* and *unc-62* mutants. Data were normalized with respect to seam cell number by comparison with transcript levels of *nuo-2*, expressed in all seam cells. Error bars are s.e.m.

CEH-20 and UNC-62 co-operate to promote asymmetric divisions in seam lineages. What downstream genetic pathway(s) do they regulate if not the Wnt pathway? *rnt-1* and *bro-1* are known regulators of seam cell proliferation and self-renewal (Kagoshima et al., 2007; Nimmo et al., 2005; Nimmo and Woollard, 2008; Xia et al., 2007), therefore we tested for regulatory interactions using *rnt-1* and *bro-1* single and double mutants. The seam cell hyperplasia (and symmetrisation of divisions) observed in *ceh-20* and *unc-62* mutants is completely suppressed in *rnt-1* and/or *bro-1* mutant backgrounds (Fig. 7A,B), suggesting that *rnt-1* and/or *bro-1* may be normally repressed by CEH-20 and UNC-62 in cells that are not destined to proliferate further. In order to test this, we measured mRNA levels using q-PCR. We found upregulation of *rnt-1* mRNA in *ceh-20* and *unc-62* single mutants, with a larger increase in double mutants (Fig. 7C). *bro-1* mRNA expression remained unchanged in these strains (Fig. 7C). Thus, the symmetrisation of seam cell divisions towards the proliferative fate in *ceh-20* and *unc-62* mutants can be explained, at least in part, by the de-repression of proliferative targets such as *rnt-1*.

### *ceh-20* and *unc-62* act redundantly with *rnt-1* and *bro-1* during early development

Intriguingly, our analysis of the *bro-1 rnt-1; unc-62* triple mutant revealed that seam cell number was slightly lower in this strain than in *bro-1 rnt-1* double mutants (Fig. 7A). This suggests a possible role for *ceh-20* and/or *unc-62* in seam cell proliferation that is redundant with *rnt-1/bro-1*. Indeed, we observed that *bro-1 rnt-1; unc-62* triple mutants and *bro-1 rnt-1; ceh-20(RNAi)* animals frequently hatched with fewer than 10 seam cells per side indicative of defects in embryonic development, a phenotype never observed in *bro-1 rnt-1* double mutants or *ceh-20*/*unc-62* mutants (supplementary material Fig. S2A). In addition triple mutants displayed high levels of embryonic and larval lethality, with synthetic lethality being even more prominent in *bro-1 rnt-1; unc-62; ceh-20(RNAi)* animals (supplementary material Fig. S2B). Reduced seam cell number is not usually correlated with lethality, suggesting that *bro-1*, *rnt-1*, *unc-62* and *ceh-20* have hitherto undescribed overlapping roles during embryogenesis and larval development, distinct from their post-embryonic roles in seam development.

### Asymmetric distribution of CEH-20 following seam cell divisions

**Fig. 8.**
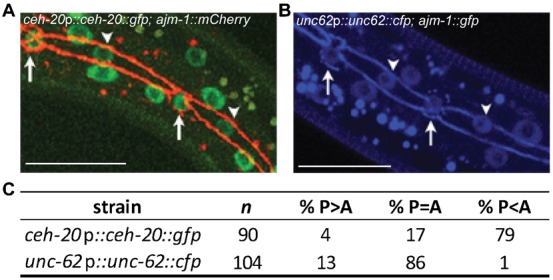
CEH-20 but not UNC-62 displays asymmetric distribution in the seam cells. (**A**) Transgenic animals carrying the rescuing *ceh-20::gfp* construct *pAW532* (strain *AW632*). Expression in seam daughters following the L3 asymmetric division is stronger in anterior nuclei that are about to differentiate (arrows), compared with posterior nuclei destined to proliferate further (arrowheads). Apical junctions surrounding seam cells were visualised using *ajm-1::mCherry*. (**B**) *unc-62::cfp* (strain *AW676*) is equally expressed in anterior (arrows) and posterior (arrowheads) daughter nuclei following asymmetric division. Note: both reporters were visualized using the CFP channel (false coloured blue). Scale bars: 20 µm. (**C**) The distribution of *ceh-20::gfp* (strain *AW632*) and *unc-62::cfp* (strain *AW676*) in seam cells at the L3 asymmetric division. The figure shows the percentage of animals with stronger expression in the posterior daughter compared to the anterior daughter (P>A), with equal expression in both daughters (P = A) and with greater expression in the anterior daughter (P<A).

During post-embryonic seam cell divisions, *rnt-1* appears to be a downstream target of CEH-20 and UNC-62, as seam cell hyperplasia is repressed in *rnt-1* mutants and *rnt-1* expression is upregulated in *ceh-20/unc-62* mutants. Thus, we were interested to test whether *ceh-20* and/or *unc-62* are differentially expressed in anterior vs posterior daughters. We used full-length *ceh-20* and *unc-62* translational reporters that were clearly observed in seam cell nuclei (Fig. 8A,B). *ceh-20::gfp* expression was observed to be higher in anterior daughters of asymmetric seam cell divisions compared with posterior daughters while no obvious differences in distribution of UNC-62::CFP were observed (Fig. 8C).

Taken together, our data suggest that CEH-20 and UNC-62 work together to repress inappropriate *rnt-1* expression during post-embryonic seam cell divisions in order to regulate the balance between proliferation and differentiation. In *ceh-20* and *unc-62* mutants this balance is lost and both daughters adopt the proliferative fate, resulting in a symmetrical division pattern.

## Discussion

The phenotypes associated with *ceh-20(mu290)* and *unc-62(ku234)* alleles are very similar, with a high level of seam cell hyperplasia, predominantly at the anterior of the worm. Lineage analysis revealed a clear cellular basis for the hyperplasia phenotype; divisions in the anterior seam cell lineages H1 and H2 are completely symmetrised towards the seam fate of further cell proliferation, giving rise to the expansion in seam cell number. In no cases were extra rounds of division observed; *ceh-20* and *unc-62* mutations only affected the symmetry of scheduled divisions, suggesting that mechanisms controlling the timing of seam cell divisions (such as the heterochronic pathway) are intact in *ceh-20* and *unc-62* mutants.

In *ceh-20; unc-62* double mutants, extensive hyperplasia was observed throughout the length of the animal. Thus, CEH-20 and UNC-62 act redundantly in V lineages, but non-redundantly in H lineages, to prevent inappropriate proliferation. This regional specialization in CEH-20/UNC-62 function may be suggestive of interaction with an additional factor. An obvious candidate for such a factor would be a Hox gene, as these are well-characterised regulators of positional identity. Indeed, Meis and Pbx proteins have been shown to act as Hox co-factors in several different systems (Shanmugam et al., 1999; Shen et al., 1999). In *C. elegans*, CEH-20 and UNC-62 form a tripartite complex with LIN-39 during vulval development (Yang et al., 2005), and with MAB-5 or LIN-39 during mesoderm development (Jiang et al., 2009). Furthermore, there is already an established link between the alternative Meis *psa-3*, Pbx (*ceh-20*) and the posterior Hox (*nob-1*) in regulating cell fate determination during the first division of the T cell. Here, *ceh-20*, *psa-3* and *nob-1* mutants have similar phenotypes, involving the failure of posterior T daughters to acquire neural fate and it has been suggested that PSA-3 and NOB-1 form a tripartite complex with CEH-20 to direct the fate choice of the posterior daughter cell (Arata et al., 2006). However, when we knocked down all six Hox genes individually by RNAi either in wild type, *ceh-20(mu290)* or *unc-62(ku234)* backgrounds we found no obvious differences in seam cell number or division pattern (S.H., unpublished), so possible interactions of CEH-20 and UNC-62 with Hox factors in H and V seam lineages remain unclear.

Although we observed that WRM-1 localisation is perturbed in *ceh-20(RNAi)* animals, *wrm-1* was clearly not required for the hyperplasia in *ceh-20(RNAi)* animals, suggesting that *ceh-20* (and presumably *unc-62*) act downstream of WRM-1, or in a parallel pathway. Furthermore, we observed that WRM-1 distribution in wild type seam cell divisions does not always follow the expected pattern; we always found WRM-1 to be asymmetrically localised between seam daughters with enrichment at the posterior nucleus, even during the L1 division of H1 (which is reversed) and the L2 symmetrical division of Vn cells. This fits with previous observations of the distribution of POP-1, which has also been reported to be asymmetric during the L2 symmetrical division (Wildwater et al., 2011). Therefore, seam cells must have some mechanism of overriding WRM-1/POP-1 asymmetry when polarity needs to be reversed, or when a symmetrical division is required.

The absolute dependence of hyperplasia in *ceh-20/unc-62* mutants on *rnt-1* but not *wrm-1* allows us to hypothesise that *ceh-20/unc-62* function in the *rnt-1* pathway, upstream of *rnt-1*, and either downstream of or in parallel to Wnt signalling. Given that *rnt-1* is thought to act in parallel to Wnt signalling (Gleason and Eisenmann, 2010), the most likely interpretation is that *ceh-20/unc-62* encode upstream regulators of *rnt-1* working in parallel to Wnt signalling to regulate seam cell divisions. This mechanism is in contrast to that described for the action of CEH-20/PSA-3/NOB-1 in the T cell, where POP-1 directly activates *psa-3* transcription in the posterior daughter of T (and no other seam cell) in conjunction with CEH-20 and NOB-1 (Arata et al., 2006). UNC-62, in contrast to PSA-3, appears to have uniform localisation throughout the seam, with no apparent bias to either daughter nucleus at division, and no obvious binding sites for POP-1 (C.B., unpublished). Overall, therefore, it is clearly not possible to directly relate this model to the more anterior lineages simply by substituting *unc-62* for *psa-3* and invoking the involvement of an anterior Hox gene.

We found that *rnt-1* expression is upregulated in *ceh-20* and *unc-62* mutants, suggesting that CEH-20/UNC-62 repress *rnt-1* expression. At present we do not know whether this repression is direct or indirect. The asymmetric expression of *ceh-20* in seam daughters suggests a model in which CEH-20 and UNC-62 repress *rnt-1* in anterior daughters destined not to proliferate further. In support of this, *rnt-1* is known not to be normally expressed in anterior daughters such as hyp7 and can be observed to disappear at or soon after division (Kagoshima et al., 2007). Forced overexpression of *rnt-1*, on the other hand, is known to cause seam hyperplasia due to inappropriate symmetrisation of seam divisions (Kagoshima et al., 2007), thus it is crucial to tightly regulate *rnt-1* expression in order to maintain the correct pattern of seam cell divisions. *ceh-20* and *unc-62* define an important mechanism to achieve this.

Interactions between *rnt-1*, *bro-1*, *ceh-20* and *unc-62* appear to be rather different during embryonic development, however, with high levels of embryonic and larval lethality in triple and quadruple mutants. This suggests redundant roles for these factors during embryogenesis, likely outside of the seam cells. Possible functions of *rnt-1* and *bro-1* during embryogenesis are unknown at present, although it is intriguing that synthetic lethality between *rnt-1* or *bro-1* and a diverse collection of developmental genes have been reported, including *dpy-22* (Xia et al., 2007), *lon-1* (Ji et al., 2004), *pha-2* and *eat-3* (Mörck and Pilon, 2006). This suggests that *bro-1* and *rnt-1* may function in several different tissues in combination with other factors.

Overall, it is striking that many of the genes isolated in unbiased screens for regulators of seam cell development have human homologues implicated in various cancers. In particular, Pbx and Meis proteins and Runx and CBFβ, are all known to regulate the proliferative potential of haematopoietic stem cells (Chen et al., 2009; Okuda et al., 1996; Thorsteinsdottir et al., 2001; Wang et al., 2006). The strong connections between perturbations in asymmetric cell divisions and tumourigenesis, underscored here in the context of hyperplasia of the stem-like seam cells, highlight the usefulness of the *C. elegans* seam cell model for defining novel elements and interactions of these pathways in a system that is not hampered by genetic redundancy experienced in systems with multiple paralogues of Runx, Pbx and Meis.

## Supplementary Material

Supplementary Material
