# Supplementary material for: CEH-20/Pbx and UNC-62/Meis function upstream of rnt-1/Runx to regulate asymmetric divisions of the C. elegans stem-like seam cells
Source: Biol Open. 2013 Jun 6;2(7):718–27. doi: 10.1242/bio.20134549 (PMC3711040; doi:10.1242/bio.20134549)
Supplement: Supplementary Material [file supp_2_7_718__index.html]

CEH-20/Pbx and UNC-62/Meis function upstream of rnt-1/Runx to regulate asymmetric divisions of the C. elegans stem-like seam cells — CEH-20/Pbx and UNC-62/Meis function upstream of rnt-1/Runx to regulate asymmetric divisions of the C. elegans stem-like seam cells — Supplementary Material 

# CEH-20/Pbx and UNC-62/Meis function upstream of *rnt-1*/Runx to regulate asymmetric divisions of the *C. elegans* stem-like seam cells

## 

**Files in this Data Supplement:**

- Supplementary Material - Samantha Hughes et al. doi: 10.1242/bio.20134549
