# Supplementary material for: CEH-20/Pbx and UNC-62/Meis function upstream of rnt-1/Runx to regulate asymmetric divisions of the C. elegans stem-like seam cells
Source: Biol Open. 2013 Jun 6;2(7):718–27. doi: 10.1242/bio.20134549 (PMC3711040; doi:10.1242/bio.20134549)
Supplement: Supplementary Material [file supp_bio.20134549_bio.20134549-s1.pdf]

Supplementary Material

Samantha Hughes et al. doi: 10.1242/bio.20134549

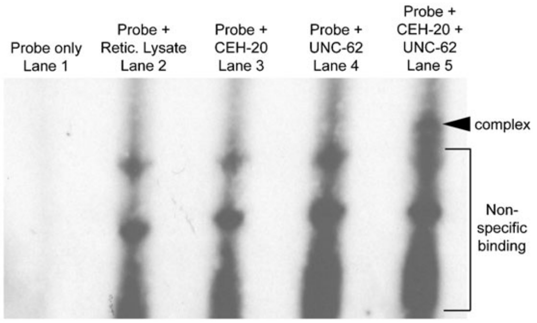

Fig. S1. UNC-62 and CEH-20 cooperatively bind to a consensus Meis/Pbx DNA binding site. Electromobility Shift Assay (EMSA) showing that *in vitro* translated UNC-62 or CEH-20 do not bind significantly above background to a consensus Pbx/Meis target sequence (lanes 3,4), whereas a complex of CEH-20 and UNC-62 retards the migration of labelled probe (lane 5, black arrowhead).

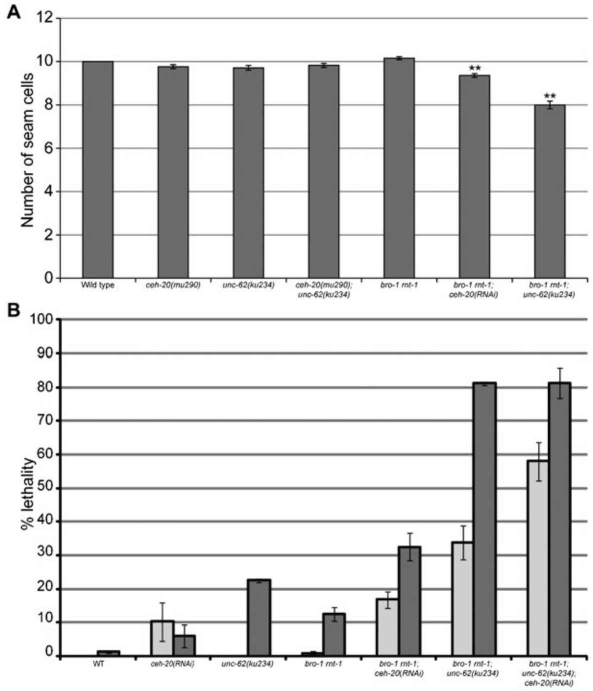

Fig. S2. *ceh-20/unc-62* and *rnt-1/bro-1* have redundant roles in embryonic development. (**A**) Seam cell numbers were counted at hatching, prior to any seam divisions taking place. *scm::gfp* animals have 10 seam cells per side (strain *JR667*; *n*=24) as do *ceh-20(mu290)* (strain *AW417*; *n*=47), *unc-62(ku234)* (strain *AW673*; *n*=52), *ceh-20;unc-62* mutants (strain *AW679*; *n*=40) and *bro-1(tm1183) rnt-1(tm388)* animals (strain *AW302*; *n*=57). In contrast *bro-1(tm1183) rnt-1(tm388); unc-62(ku234)* animals (strain *AW679*; *n*=34) and *bro-1(tm1183) rnt-1(tm388); ceh-20(RNAi)* animals (*n*=78) have 8 and 9 seam cells per side respectively at L1. Error bars represent the s.e.m. where ** indicates $P<0.01$. (**B**) Graph to show embryonic lethality (light grey bars) and larval lethality of those larvae that survived embryonic development (dark grey bars). Strains are indicated on the x-axis. Error bars represent the s.e.m., $n>100$ eggs laid for each strain analysed.

Biology Open

Biology Open

Table S1. Strains used. List of the strains used with transgenic arrays detailed.

Strain	Genotype	Plasmids in extrachromosomal arrays
JR667	*unc-119(e2498)*III; *wIs51[pMF1+pDP#MM016β]* V	*pMF1[scm::gfp]*<br>*pDP#MM016β[unc-119(+)]*
AW186	*bro-1(tm1183)*I; *him-5(e1490)wIs51[pMF1+pDP#MM016β]* V	*pMF1[scm::gfp]*<br>*pDP#MM016β [unc-119(+)]*
AW187	*rnt-1(tm388)*I*; him-5(e1490)wIs51[pMF1+pDP#MM016β]* V	*pMF1[scm::gfp]*<br>*pDP#MM016β[unc-119(+)]*
AW302	*bro-1(tm1183) rnt-1(tm388)*I*; him-5(e1490)wIs51[pMF1+pDP#MM016β]* V	*pMF1[scm::gfp]*<br>*pDP#MM016β[unc-119(+)]*
AW392	*unc-62(e644)*V; *him-5(e1490)wIs51[pMF1+pDP#MM016β]* V	*pMF1[scm::gfp]*<br>*pDP#MM016β[unc-119(+)]*
AW413	*ceh-20(ay9)*III; *him-5(e1490)wIs51[pMF1+pDP#MM016β]* V	*pMF1[scm::gfp]*<br>*pDP#MM016β[unc-119(+)]*
AW417	*ceh-20(mu290)*III; *ouEx583[pMF1]*	*pMF1[scm::gfp]*
AW423	*unc-119(ed3)*III; *him-8(e1489)*IV; *ouEx123[pAW260+pAW526+pPD#MM016β]*	*pAW260 [rnt-1*p*:rnt-1:gfp]*<br>*pAW525 [ajm-1::mCherry]*<br>*pDP#MM016β[unc-119(+)]*
AW525	*unc-119(ed3)*III*; syIs78; arIs99*	*syIs78[ajm-1::gfp+unc-119(+)]*<br>*arIs99[dpy-7p::2xnls::yfp]*
AW541	*unc-119(ed3)*III*; ouEx223[pAW537+pDP#MM016β]*	*pAW537[bro-1CNE::ceh-20(mu290)::gfp]*<br>*pDP#MM016β[unc-119(+)]*
AW550	*unc-119(ed3)*III*; ouEx232[pAW551+pDP#MM016β]*	*pAW551[bro-1CNE::ceh-20(ay9)::gfp]*<br>*pDP#MM016β[unc-119(+)]*
AW555	*unc-119(ed3)*III*; ouEx235[pAW538+pDP#MM016β]*	*pAW538[bro-1CNE::ceh-20(WT)::gfp]*<br>*pDP#MM016β[unc-119(+)]*
AW564	*unc-62(ku234)*V*; ouEx243[pAW538+pAW378]*	*pAW538[bro-1CNE::ceh-20(WT)::gfp]*<br>*pAW378 [unc-122::rfp]*
AW593	*unc-119(ed3)*III; *ouEx566[pAW588+pDP#MM016β]*	*pAW588[bro-1CNE::ceh-20(*mutated NLSI*)::gfp]*<br>*pDP#MM016β[unc-119(+)]*
AW601	*unc-119(ed3)*III*; ouEx574[pAW589+pDP#MM016β]*	*pAW589[bro-1CNE::ceh-20(*mutated NLSI+*mu290)::gfp*<br>*pDP#MM016β[unc-119(+)]*
AW632	*unc-119(ed3)*III; *ouEx597[pAW532+pAW526+pDP#MM016β]*	*pAW532[ceh-20p::ceh-20(WT)::gfp]*<br>*pAW526[ajm-1::mCherry]*<br>*pDP#MM016β[unc-119(+)]*
AW673	*unc-62(ku234)*V; *him-5(e1490)wIs51[pMF1+pDP#MM016β]* V	*pMF1[scm::gfp]*<br>*pDP#MM016β[unc-119(+)]*
AW674	*bro-1(tm1183) rnt-1(tm388)*I*; unc-62(ku234)*V*; him-5(e1490)wIs51[pMF1+pDP#MM016β]*V	*pMF1[scm::gfp]*<br>*pDP#MM016β[unc-119(+)]*
AW676	*unc-119(ed3)*III*; syIs78[pMP062+pJS191+pDP#MM016β]*	*pMP062[unc-62p::unc-62::cfp*]<br>*pJS191[ajm-1::gfp*]<br>*pDP#MM016β[unc-119(+)]*
AW679	*unc-62(ku234)*V; *ceh-20(mu290)*III; *him-5(e1490)wIs51[pMF1+pDP#MM016β]* V	*pMF1[scm::gfp]*<br>*pDP#MM016β[unc-119(+)]*
AW682	*unc-62(ku234)V; arIs99 X*	*arIs99[dpy-7p::2xnls::yfp]*
EW95	*wrm-1(ne1982*ts*); pMF1*	*pMF1[scm::gfp]*
HS1417	*unc-72(e911)*V; *osIs5[scm::wrm-1::gfp+punc-76^{+}]*II	

Table S2. Primer pairs used. Details of each primer pair used in this paper. Primers are written 5′-3′.

Primer pair	Forward primer 5′-3′	Reverse primer 5′-3′
1	GAATGATGGAAATCCACCGTAC	ACGTCAACTCCGATGATGC
2	ATGGTGGGGACACACCCAG	CGACCTGCAGGCATGCAAGCTTGAATTTTTATCAGCATTGTTCC
3	GCTTGCATGCCTGCAGGTCG	AAGGGCCCGTACGGCCGACTAGTAGG
4	ATGGTGGGGACACACCCAG	AAACAGTTATGTTTGGTATATTGGG
5	CCCGCATGCGAGAAACTCGGTGCATTCTTGCATG	TGCCTGCAGGAAACAAAGAAGAGGAAATGACCTTGAATTAG
6	CCGCGGCGAAACTTTTCG	ACGGCGGGCATCAAGG
7	ATGGTGGGGACACACCCAG	GGTTGAGATTTCAATATTACCAACTGGCTG
8	GAATGATGGAAATCCACCGTAC	ACGTCAACTCCGATGATGC
9	CAAGGTTACGGTTGATGGACCAAGG	CATTGGCATCGGTGGTCCAGG
10	ATTGAAAACCAGTGGCCGAGAAC	TGAAATGCCCTTGACTTTCTGCAAG
11	GGTGCTGACTGGTTTGAGCG	TCCTTGCGGAATGGGTGACC

Biology Open

Table S3. List of genes isolated from the genome wide RNAi screen for regulators of seam cell number. A list of genes (307) which, when silenced, result in a change in seam cell number.

Fewer seam cells		More seam cells	
Gene ID	Gene name	Gene ID	Gene name
M01D7.6	*emr-1*	B0414.8	*B0414.8*
F53G12.11	*F53G12.11*	D1007.6	*rps-1*
R12E2.3	*rpn-8*	D1007.2	*col-52*
R119.7	*rnp-8*	B0414.8	*B0414.8*
C55C2.2	*ssp-19*	F27C1.10	*F27C1.10*
T22E7.2	*T22E7.2*	F56E7.1	*F56E7.1*
C24A11.9	*coq-1*	W01A8.4	*W01A8.4*
Y110A7A.4	*Y110A7A.4*	F27D4.1	*F27D4.1*
M01A10.1	*M01A10.1*	K10D3.3	*taf-11.2*
C48E7.2	*C48E7.2*	F13G3.10	*F13G3.10*
F20G4.1	*smgl-1*	F46F11.4	*ubl-5*
F30F8.8	*taf-5*	Y54E10A.12	*Y54E10A.12*
K07A1.12	*lin-53*	T02E1.2	*T02E1.2*
F26E4.4	*F26E4.4*	ZK858.2	*ZK858.2*
B0511.3	*fbxa-125*	M04C9.1	*M04C9.1*
B0511.4	*tag-344*	F46A9.6	*mec-8*
B0511.6	*B0511.6*	F26E4.11	*hrdl-1*
C17D12.2	*unc-75*	C34B2.9	*C34B2.9*
K05C4.1	*pbs-5*	T22H2.3	*sri-11*
F56A3.4	*spd-5*	E03H4.3	*E03H4.3*
Y110A7A.14.1	*pas-3*	C01A2.4	*C01A2.4*
F59A3.2	*F59A3.2*	Y18D10A.5	*gsk-3*
M01E11.2	*M01E11.2*	Y34D9A.6	*glrx-10*
F56F4.2	*ttr-55*	C49A1.1	*C49A1.1*
T10E9.7	*nuo-2*	Y34D9A_150.a	*Y34D9A_150.a*
F37E3.1	*ncbp-1*	K04F10.3	*K04F10.3*
R06C7.6	*R06C7.6*	C26C6.7	*glb-8*
M04C7.2	*M04C7.2*	F13G3.3	*F13G3.3*
R05D11.3	*ran-4*	T28B8.4	*T28B8.4*
R13H8.1	*daf-16*	T01G9.6	*kin-10*
F55A3.1	*.marc-6*	F02E9.4	*sin-3*
F55A3.2	*F55A3.2*	F02E9.5	*F02E9.5*
F36D1.2	*sre-22*	K07A12.5	*K07A12.5*
Y18D10A.13	*pad-1*	T04D3.4	*gcy-33*
Y105E8C.8	*Y105E8C.8*	C49A1.9	*clec-114*
F31C3.2	*F31C3.2*	F12B6.3	*lgc-51*
W03D8.3	*W03D8.3*	W10C8.2	*pop-1*
F47B3.3	*F47B3.3*	W07E6.6	*W07E6.6*
C18E3.6	*cas-2*	W08F4.8	*cdc-37*
C08E3.5	*fbxa-162*	K07E8.3	*sdz-24*
F16G10.7	*F16G10.7*	F58E1.10	*fxbc-22*
F16G10.14	*F16G10.14*	C16C4.14	*math-9*
F33H12.5	*sri-36*	W09G10.5	*clec-126*
F58E1.13	*F58E1.13*	C01F1.1	*C01F1.1*
M151.6	*FBXB-32*	F54D10.3	*F54D10.3*
C33F10.10	*C33F10.10*	C34F11.1	*C34F11.1*
E04F6.11	*clh-3*	F09E5.8	*F09E5.8*
K10C8.10	*K10C8.10*	K10B2.5	*ani-2*
M195.1	*col-77*	C28F5.4	*C28F5.4*
D2085.3	*D2085.3*	C28F5.5	*C28F5.5*
D2013.7	*eif-3.F*	F59F12.3	*F59F12.3*
C06C3.4	*C06C3.4*	C56E6.3	*toe-2*
F54D5.11	*F54D5.11*	B0495.5	*B0495.5*
M28.6	*lact-3*	C06A8.5	*spdl-1*
F33H1.5	*srd-1*	T09A5.11	*T09A5.11*
T01E8.3	*plc-3*	T05H10.2	*apn-1*
C50E10.6	*sre-54*	T05H10.4	*T05H10.4*
W01D2.2	*nhr-61*	C41C4.1	*C41C4.1*
W01D2.3	*W01D2.3*	C41C4.2	*sre-2*
W03H9.4	*cacn-1*	C15F1.4	*ppp-1*
Y48B6A.14	*hmg-1.1*	T07F8.1	*T07F8.1*
R06A4.10	*acr-20*	F10B5.6	*emb-27*
K10H10.2	*K10H10.2*	F15A4.3	*sre-37*
C01G12.6	*nspb-10*	K02B7.3	*K02B7.3*
F54A3.1	*F54A3.1*	C09F9.2	*C09F9.2*
Y46G5A.2	*Y46G5A.2*	F01D5.6	*F01D5.6*
Y46G5.i	*Y46G5.i*	Y48B6A.7	*ace-4*
Y51H7C.13	*Y51H7C.13*	E01G4.5	*E01G4.5*

Table S3. Continued.

Fewer seam cells		More seam cells	
**Gene ID**	**Gene name**	**Gene ID**	**Gene name**
F54C4.3	*F54C4.3*	F45H7.6	*F45H7.6*
F40G9.1	*F40G9.1*	C36A4.5	*C36A4.5*
C54C6.5	*C54C6.5*	C27F2.8	*C27F2.8*
C34E10.2	*gop-2*	K10D2.7	*K10D2.7*
R07E5.14	*rnp-4*	R74.5	*asd-1*
F35G12.8	*smc-4*	R07E5.10	*pdcd-2*
T04A8.6	*T04A8.6*	F31E3.1	*ceh-20*
C16C10.2	*C16C10.2*	T04A6.3	*T04A6.3*
C05D11.9	*C05D11.9*	C02C2.3	*cup-4*
F37C12.1	*F37C12.1*	K03H1.11	*K03H1.11*
F37C12.13	*exos-9*	ZK1128.2	*ZK1128.2*
T20B12.1	*T20B12.1*	ZC482.5	*lgc-37*
C07H6.1	*lig-4*	Y37D8A.16	*Y37D8A.16*
ZK112.6	*ZK112.6*	F29C4.8	*col-99*
ZK652.9	*coq-9*	F36A4.2	*F36A4.2*
F44B9.2	*F44B9.2*	F45E4.11	*F45E4.11*
C50C3.9	*unc-36*	F57H12.1	*arf-3*
K02D10.3	*K02D10.3*	F13B12.3	*F13B12.3*
ZK512.5	*ZK512.5*	T25B9.10	*T25B9.10*
B0464.4	*bre-3*	ZK593.3	*ZK593.3*
K01G5.1	*rnf-113*	Y5F2A.3	*Y5F2A.3*
Y47D3B.2	*nlp-21*	R07H5.1	*prx-14*
Y111B2b.a	*Y111B2b.a*	M04B2.3	*gfl-1*
BE10.2	*BE10.2*	ZC518.3	*ccr-4*
Y43F4A.4	*Y43F4A.4*	JC8.6	*lin-54*
Y43F4B.5	*Y43F4B.5*	Y37A1B.11	*egl-23*
Y66H1A.1	*Y66H1A.1*	H12I19.5	*oac-38*
M04G7.1	*M04G7.1*	Y55F3AM.6	*Y55F3AM.6*
Y38C1BA.2	*snn-1*	F22F7.5	*ckb-4*
C04C3.6	*C04C3.6*	F15E11.10	*sbrc-15*
ZK354.7	*ZK354.7*	C24B9.10	*srg-56*
C17H12.6	*C17H12.6*	F54E2.3	*ketn-1*
T05A12.4	*T05A12.4*	F41F3.6	*srx-74*
T09A12.4	*nhr-66*	T05C3.2	*T05C3.2*
F42G8.3	*pmk-2*	T05H4.5	*T05H4.5*
D2096.5	*D2096.5*	T05H4.12	*atp-4*
D2096.6	*D2096.6*	T15B7.10	*T15B7.10*
T26A8.2	*T26A8.2*	T15B7.16	*lgc-54*
H23L24.4	*H23L24.4*	F25G6.5	*stdh-4*
T28C6.1	*grsp-2*	B0222.2	*B0222.2*
D1046.5	*D1046.5*	F40F9.5	*F40F9.5*
ZC410.4	*twk-8*	F17C11.5	*clec-221*
F56D5.7	*F56D5.7*	C13G3.3	*pptr-2*
F56D5.10	*srxa-2*	T19C4.4	*srg-40*
F32B6.4	*F32B6.4*	F07B10.3	*str-115*
C04H5.6	*mog-4*	F25E5.9	*F25E5.9*
R13.1	*miz-1*	R10D12.3	*srx-127*
C08F8.1	*pfd-1*	C25D7.10	*C25D7.10*
ZK809.5	*ZK809.5*	F36D3.1	*F36D3.1*
F52B11.3	*noah-2*	T03E6.1	*str-151*
F52B11.5	*F52B11.5*	Y51A2D.15	*Y51A2D.15*
F38C2.5	*ccch-2*	F11D11.3	*F11D11.3*
Y51H4A.25.a.1	*Y51H4A.25.a.1*	B0310.6	*B0310.6*
Y37E11A_93.c	*Y37E11A_93.c*	F56F10.4	*F56F10.4*
Y41D4B.19b	*npp-8*	EGAP7.1	*dpy-3*
Y46C8_98.b	*Y46C8_98.b*	T24D8.5	*nlp-2*
Y9C9A_54.c	*Y9C9A_54.c*	F15A8.2	*F15A8.2*
F56E10.2	*fhod-2*	K07E3.2	*K07E3.2*
B0554.8	*B0554.8*	F55E10.2	*F55E10.2*
K06H6.5	*K06H6.5*	D2021.1	*utx-1*
ZK488.10	*pqn-97*	ZK721.2	*unc-27*
C05E4.12	*C05E4.12*	F44A6.2	*sex-1*
K09C6.3	*K09C6.3*	F41E7.6	*F41E7.6*
C45H4.17	*cyp-33C2*	F11C1.6	*nhr-25*
F44C8.1	*cyp-33C4*	F46G10.6	*mxl-3*
F26F2.4	*F26F2.4*	C31E10.5	*C31E10.5*
Y39B6A.7	*Y39B6A.7*	C11H1.5	*C11H1.5*
Y61A9LA.5	*Y61A9LA.5*	F59C12.2	*ser-1*
C04E12.9	*srbc-2*	C06G1.5	*C06G1.5*

Fewer seam cells	
**Gene ID**	**Gene name**
T24A6.1	*T24A6.1*
M03F8.3	*phi-12*
C13A2.9	*C13A2.9*
C12D5.9	*C12D5.9*
ZC513.1	*ZC513.1*
F20D6.10	*F20D6.10*
F55A11.2	*syn-3*
F55B12.4	*F55B12.4*
ZK863.6	*dpy-30*
C56A3.2	*ttr-44*
R08H2.8	*R08H2.8*
K06B4.10	*nhr-199*
C31A11.9	*str-214*
Y32B12A.2	*srbc-73*
T03E6.6	*swr-73*
Y68A4A.7	*srh-113*
Y59A1.11	*Y59A1.11*
C31G12.1	*C31G12.1*
Y116F11A.b	*Y116F11A.b*
Y113G7B.21	*mdt-17*
F19B2.7	*F19B2.7*
ZK1193.5	*dve-1*
Y59E1A.1	*FBXA-40*
F49H12.1	*lsy-2*
R02E12.8	*acr-10*
ZK470.1	*ZK470.1*
F13D11.2	*hbl-1*
F08C6.1	*adt-2*
C18A11.5	*xol-1*
H35N09.1	*H35N09.1*
F47B10.1	*F47B10.1*
C30F2.1	*col-187*
F22H10.2	*F22H10.2*

Biology Open
